# Exploring the Synergy Between Language Semantic Guidance and Visual Attention in Knowledge Distillation for Semantic Segmentation Under a Limited Field of View

**DOI:** 10.3390/biomimetics11090665

**Published:** 2026-09-16

**Authors:** Jin Hyeok Ryu, Seung Woo Cha, Eun Som Jeon

**Affiliations:** Department of Computer Science and Engineering, Seoul National University of Science and Technology, Seoul 01811, Republic of Korea; ryujin0402@seoultech.ac.kr (J.H.R.); tmddn4182@seoultech.ac.kr (S.W.C.)

**Keywords:** semantic segmentation, knowledge distillation (KD), attention mechanism, semantic guidance, large language models (LLMs), vision-language learning, autonomous driving, limited field-of-view (FoV)

## Abstract

Semantic segmentation for autonomous driving is challenged by limited field-of-view (FoV), where occlusions and restricted viewpoints reduce available visual information. Existing approaches often incur considerable computational overhead, which is unsuitable for autonomous driving systems. To build robust and lightweight semantic segmentation models, attention-based knowledge distillation (KD) can be an efficient alternative. Nevertheless, KD remains primarily visual and provides limited information when visual cues are incomplete or ambiguous. In contrast, biological visual systems integrate visual cues with contextual and semantic knowledge to support robust scene perception. Motivated by this biomimetic principle, language-guided methods provide high-level semantic knowledge, offering another promising direction for enhancing visual understanding. However, the effectiveness of attention-based KD under limited FoV conditions and its interaction with semantic knowledge remain largely unexplored. To systematically investigate this problem, we devise Semantic-guided Attentive Feature Distillation (SAFD), a KD-based framework that combines attention-based feature distillation with semantic guidance derived from large language models (LLMs) without introducing additional inference-time computation. Through comprehensive analyses of multiple distillation strategies and textual representations using the framework, we observe that semantic guidance generally provides additional benefits when the gains from visual distillation are limited, while its effectiveness varies across different attention-based distillation strategies and textual representations. Furthermore, our analyses reveal that different attention mechanisms exploit semantic guidance in distinct ways, with consistent benefits across different pretrained text encoders and improved generalization. These findings provide practical insights into integrating linguistic semantics with visual knowledge transfer under limited FoV.

## 1. Introduction

Semantic segmentation is a key component in autonomous driving systems, enabling pixel-level understanding of scenes by distinguishing objects such as roads, vehicles, and pedestrians. This fine-grained perception is fundamental to ensuring safe and reliable decision-making. Consequently, recent advances in deep learning have significantly improved the performance of semantic segmentation for autonomous driving [1,2]. However, most existing studies assume a wide and unobstructed field of view (FoV), which is an idealized setting where the full context of a driving scene is available [3,4]. In real-world scenarios, the camera view is often constrained by its physical position, viewing angle, or occlusions caused by surrounding objects. Such limited FoV conditions can lead to the loss of contextual information, thereby degrading the segmentation performance [5,6].

Although restoring missing visual regions [7] or leveraging multiple inputs [8] can mitigate this issue, these methods significantly increase computational overhead. While larger models can generally accommodate such heavy resource demands, they are unsuitable for the real-time requirements of autonomous driving systems. Therefore, achieving a balance between performance and computational efficiency remains a critical challenge.

In this light, knowledge distillation (KD), an effective approach for obtaining a lightweight model (student) by transferring knowledge from a large high-capacity model (teacher), has been widely adopted [9]. KD methods can be categorized into response-based, feature-based, and relation-based approaches [10]. Among feature-based methods, attention-based feature distillation methods [11,12,13] have demonstrated promising performance by selectively emphasizing informative spatial regions or feature channels for more effective visual knowledge transfer. These attention-based methods have also shown strong performance in semantic segmentation tasks [14,15]. However, despite this effectiveness, these distillation methods remain inherently dependent on visual information, making it difficult to compensate for missing contextual cues under limited FoV conditions.

To mitigate this loss of contextual information, an alternative approach has been developed to improve the model’s semantic understanding. This direction can be viewed as conceptually related to biological visual systems, where scenes and objects are perceived by integrating diverse information, such as visual information with contextual and semantic knowledge [16]. Cognitive studies demonstrate that such knowledge is represented as statistical summaries [17] or semantic relations within a scene [18]. Inspired by this functional principle, recent bio-inspired vision systems have integrated complementary multimodal cues [19] and high-level semantic information [20] into perceptual pipelines to achieve more comprehensive and robust visual perception.

Building on this biomimetic perspective, linguistic information can serve as an effective medium for providing computer vision models with semantic priors that function similarly to the high-level knowledge supporting biological scene perception [21,22,23]. More recently, large language models (LLMs) have attracted increasing attention because they provide richer semantic knowledge through their expressive language generation capabilities [24,25]. Building upon such semantic knowledge, vision-language models (VLMs) have been utilized to provide vision-aligned semantic priors that capture contextual relationships often missing from purely visual observations [26,27]. However, most existing approaches [28,29] require additional linguistic inputs during inference, which increases model complexity and limits their practicality for deployment on resource-constrained edge devices.

Despite the progress of these two research directions, two key issues remain unresolved. First, although attention-based feature distillation has demonstrated promising performance in conventional semantic segmentation, its effectiveness under limited FoV conditions has not been sufficiently investigated. Second, while linguistic semantic knowledge and attention-based visual distillation offer complementary sources of supervision, their interaction during knowledge distillation remains poorly understood. Motivated by these gaps, this paper aims to investigate the following questions: *(i) Can semantic knowledge derived from LLMs complement the visual knowledge transferred by attention-based KD without introducing inference-time overhead? (ii) How do different attention-based KD strategies and textual representations jointly influence semantic segmentation performance under limited FoV conditions?*

To investigate these questions, we design a knowledge distillation framework, termed Semantic-guided Attentive Feature Distillation (SAFD), which combines attentive vision-based feature distillation with LLM-derived knowledge as semantic guidance. From a biomimetic perspective, SAFD draws on the principle of context-assisted biological perception by using language-derived semantic priors to complement incomplete visual cues within a lightweight learning framework. Importantly, this semantic guidance is incorporated only during training as auxiliary supervision, introducing no additional computational overhead during inference. Furthermore, through a comprehensive exploration of various attention-based visual distillation strategies and textual representations within the unified SAFD framework, we analyze their interactions and synergistic effects under limited FoV conditions.

Our main contributions are summarized as follows:

- We devise a SAFD framework that incorporates LLM-derived semantic knowledge into feature distillation without increasing inference complexity.
- We systematically investigate multiple attention-based visual feature distillation strategies and textual representations to analyze how semantic guidance interacts with different forms of visual supervision.
- We show that semantic guidance is especially effective when visual distillation provides limited semantic supervision, while its effectiveness depends on the visual representations transferred from the teacher, the adopted attention strategy, and the dataset characteristics.
- We further demonstrate that semantic guidance produces distinct class-wise confusion patterns across attention-based distillation strategies and consistent performance gains across the evaluated text encoders, while appropriate combinations with each attention strategy can improve generalizability and prediction reliability.

## 2. Related Work

### 2.1. Semantic Segmentation in Limited FoV

The challenge of semantic segmentation in road scenes with limited FoV has been addressed in several ways [7,8,30,31]. Shi et al. [7] and Ma et al. [30] focus on improving semantic segmentation performance by jointly performing image outpainting and semantic segmentation to recover missing visual information beyond the FoV. These methods rely on outpainting restoration to supplement the lack of visual information under limited FoV conditions, which leads to additional computational latency and makes them less practical for real-time autonomous driving systems. In addition, multi-FoV approaches have been proposed to broaden the field of view and extract richer visual features through multiple image matching [8]. Furthermore, Seifi et al. [31] proposed an active segmentation framework in which an agent sequentially explores different regions and infers segmentation for unobserved areas via a self-supervised attention mechanism. Although these approaches effectively recover invisible regions, they still require additional computation, limiting their applicability in autonomous driving scenarios.

### 2.2. Knowledge Distillation

KD has attracted significant interest as an effective approach for improving semantic segmentation while maintaining computational efficiency [9]. KD enables a compact student model to learn informative knowledge from a larger teacher model, thereby facilitating the design of lightweight yet high-performing models. Existing KD methods are commonly grouped into response-based [9,32], feature-based [11,33,34], and relation-based distillation [35,36,37]. Among feature-based KD methods, attention mechanisms have been widely used to transfer refined feature representations by emphasizing informative spatial regions or feature channels, thereby improving the effectiveness of feature transfer [11,12,13,15,38]. Specifically, Attention Transfer (AT) [11] computes spatial attention maps by summing absolute activations across the channel dimension. Channel Distillation (CD) [38] employs channel attention by matching globally pooled channel responses between the teacher and student. Attention Similarity Knowledge Distillation (A-SKD) [13] and Attention-guided Feature Distillation (AttnFD) [15] both employ the Convolutional Block Attention Module (CBAM) [39] to jointly exploit spatial and channel attention for feature distillation. These studies demonstrate that attention mechanisms effectively improve feature transfer by selectively highlighting spatial structures and channel representations. Although these attention-based KD methods have demonstrated remarkable performance, the effectiveness of their spatial or channel attention has not been sufficiently explored for semantic segmentation under limited FoV scenarios.

### 2.3. Language-Guided Semantic Segmentation

Recent advances in vision-language learning have enabled language information to be incorporated into various vision tasks [21,22,23]. In particular, LLMs [40,41,42] have recently been employed to generate textual descriptions or semantic priors for visual recognition [24,25]. Building upon these advances, language-guided semantic segmentation methods leverage language as an additional semantic cue to enrich visual scene understanding [23,43,44]. Existing approaches [28,29] incorporate language into semantic segmentation by jointly exploiting image and text features for modeling inter-class relationships and employing language to capture socially defined semantics that cannot be inferred solely from the physical appearance of objects. Despite their effectiveness, these methods typically require large vision-language backbones or additional linguistic inputs during inference, resulting in increased computational overhead. Moreover, multimodal feature extraction and fusion further increase computational complexity, which is unsuitable for real-time systems such as autonomous driving.

## 3. Visual Attention and Semantic Guidance Strategies

In this section, we introduce a framework that leverages visual attention-based KD and language guidance to improve the student’s semantic understanding during training without inference-time overhead. We employ different attention-based distillation strategies and different types of linguistic representations to identify the relationships between them in the training of the student model.

### 3.1. Visual Attention for Knowledge Transfer

To transfer informative visual knowledge from a teacher model, we employ several distillation strategies inspired by Attention-guided Feature Distillation (AttnFD) [15]. AttnFD leverages the Convolutional Block Attention Module (CBAM) [39], which considers both spatial and channel-wise attention for semantic segmentation distillation. This acts as a refinement to reduce noise and emphasize useful information to transfer informative knowledge. Motivated by the effectiveness of attention-based feature refinement, we employ three different approaches: (1) channel attention, (2) spatial attention, and (3) their sequential combination (channel + spatial) that is similar to CBAM. All refinement approaches share the same loss function:

(1) $$\begin{array}{c} L_{VisKD}=∥\frac{{\tilde{F}}_{S}}{∥{\tilde{F}}_{S}∥}-\frac{{\tilde{F}}_{T}}{∥{\tilde{F}}_{T}∥}∥, \end{array}$$

where ${\tilde{F}}_{S}$ and ${\tilde{F}}_{T}$ represent the refined feature maps of the student and teacher models, respectively. Before computing the distillation loss, each feature map is normalized along the channel dimension. Under this unified loss, the refinement mechanism is instantiated with one of the following three attention modules.

- **Channel Attention Module (CAM):** Woo et al. [39] proposed two attention modules, the first of which is the Channel Attention Module (CAM) utilizing the inter-channel relationship of features by aggregating spatial information. Specifically, given an input feature map $F\in R^{C\times H\times W}$ (*C*, *H*, and *W* denote the channel, height, and width dimensions, respectively), the max-pooling and average-pooling operations are applied along the spatial dimensions to generate a channel attention map $M_{c}$, highlighting ‘what’ is meaningful in a given image. From a distillation perspective, this approach facilitates the transfer of discriminative channel-wise knowledge to the student model. Following the CAM, the channel-refined feature map ${\tilde{F}}_{c}$ is formulated as:
  (2) $$\begin{array}{cc} \; & M_{c}\left(F\right)=\sigma \left(W_{1}\left(W_{0}\left(F_{avg}^{c}\right)\right)+W_{1}\left(W_{0}\left(F_{max}^{c}\right)\right)\right),\\ \; & {\tilde{F}}_{c}=M_{c}\left(F\right)⊗F, \end{array}$$
  where $F_{avg}^{c}\in R^{C\times 1\times 1}$ and $F_{max}^{c}\in R^{C\times 1\times 1}$ represent the feature maps resulting from average-pooling and max-pooling operations on the feature map *F*, respectively. $W_{0}$ and $W_{1}$ are the weights of the multi-layer perceptron (MLP) shared for the two pooled feature maps, and $W_{0}$ is followed by a ReLU activation function. σ denotes the sigmoid function and ⊗ denotes element-wise multiplication.
- **Spatial Attention Module (SAM):** The second module is the Spatial Attention Module (SAM), which exploits the spatial relationship within the feature maps. Similar to CAM, two feature maps $F_{avg}^{s}\in R^{1\times H\times W}$ and $F_{max}^{s}\in R^{1\times H\times W}$ are generated by applying average-pooling and max-pooling operations to the feature map *F* along the channel dimension. These maps are then concatenated and passed through a convolutional layer to generate a spatial attention map $M_{s}$ highlighting ‘where’ the informative regions are. Following the same principle, the spatially refined feature map ${\tilde{F}}_{s}$ of the SAM is computed as:
  (3) $$\begin{array}{cc} \; & M_{s}\left(F\right)=\sigma \left(\mathrm{Conv}\left(\left[F_{avg}^{s};F_{max}^{s}\right]\right)\right),\\ \; & {\tilde{F}}_{s}=M_{s}\left(F\right)⊗F. \end{array}$$
- **Convolutional Block Attention Module (CBAM):** CBAM uses CAM and SAM sequentially to complement the features. Specifically, the channel-refined feature map ${\tilde{F}}_{c}$ is generated using the channel attention map $M_{c}$ and is then further refined by the spatial attention map $M_{s}$ to produce the channel- and spatially refined feature map ${\tilde{F}}_{cs}$. The dual attention process is formulated as follows:
  (4) $$\begin{array}{cc} \; & {\tilde{F}}_{c}=M_{c}\left(F\right)⊗F,\\ \; & {\tilde{F}}_{cs}=M_{s}\left({\tilde{F}}_{c}\right)⊗{\tilde{F}}_{c}. \end{array}$$

### 3.2. Semantic Knowledge Extraction

To leverage linguistic semantic knowledge and investigate the effectiveness of different textual representations, we construct three types of semantic descriptions: *Label*, *Keywords*, and *Sentence*. Each format conveys the same semantic concept through different lexical and syntactic forms.

To generate the *Keywords* and *Sentence* formats, we employ GPT-5 [42], leveraging its extensive general knowledge and sophisticated linguistic expression capabilities. Here, “cls” denotes a class label. The prompt template for the *Keywords* is defined as follows:

All classes in an urban driving scene are "{cls 1}", "{cls 2}", … "{cls N}". Tell me about the {k} general words that describe each class.

where *k* is a predefined constant set to 5 during our experiments. Similarly, the prompt template for *Sentence* format is structured as follows:

All classes in an urban driving scene are "{cls 1}", "{cls 2}", … "{cls N}". Tell me about the general sentences that describe each class.

The generated descriptions used in our experiments are summarized in Table 1.

Unlike the other formats, the *Label* format is manually constructed without an LLM. Following the widely adopted observation from CLIP that using descriptions such as “A photo of {cls}” enhances representation performance [26], we directly apply this template style to each category.

### 3.3. Semantic-Guided Attentive Knowledge Distillation

**Figure 1:**
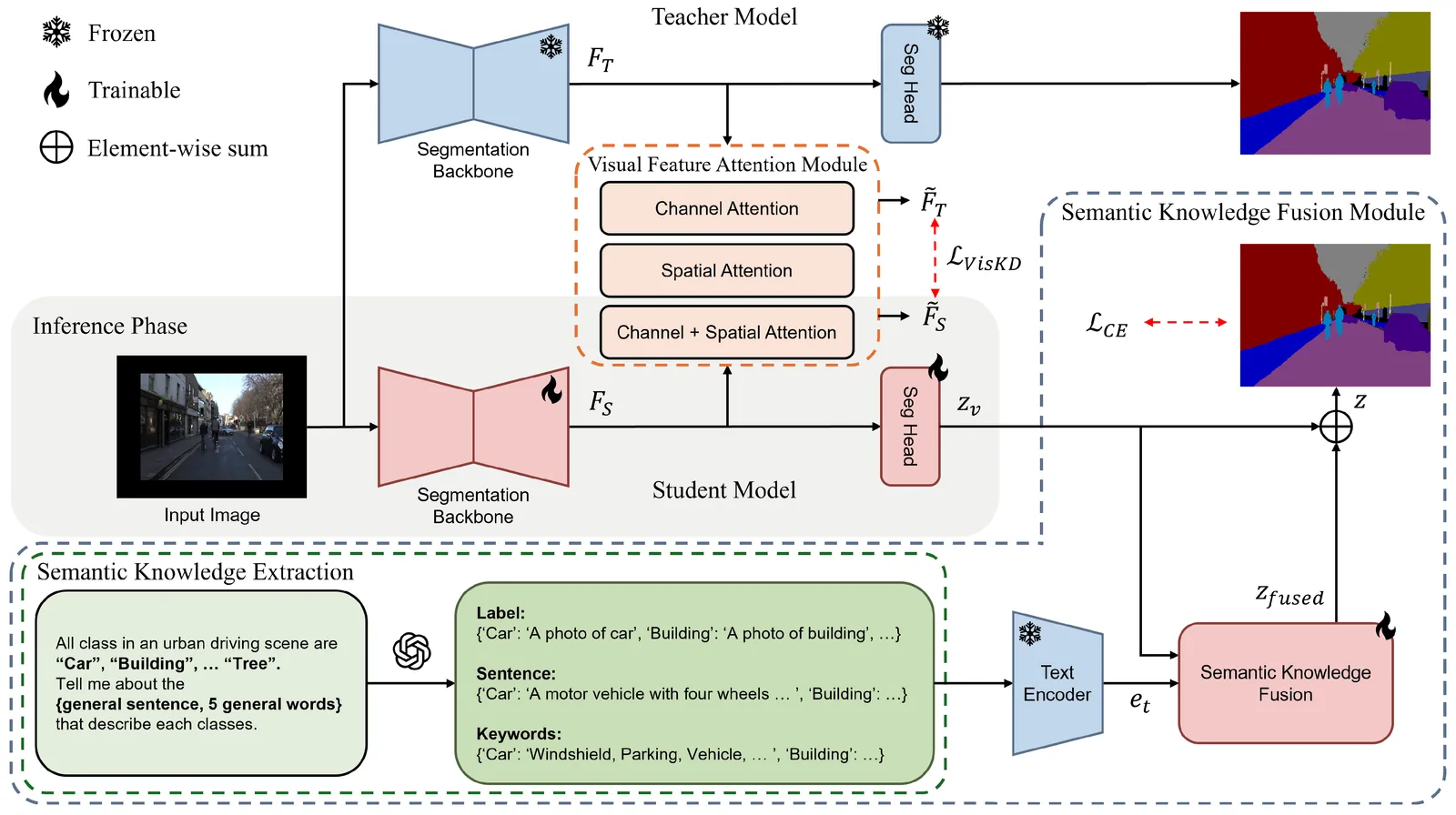
Overview of Semantic-guided Attentive Feature Distillation (SAFD) framework.

Figure 1 illustrates the overall framework, named Semantic-guided Attentive Feature Distillation (SAFD), which incorporates visual attention mechanisms and language guidance. SAFD consists of two main components during the training phase: the visual feature attention module and the semantic knowledge fusion module.

**Visual Feature Attention Module.** In the feature transfer process, the student model leverages attentive visual representations from the teacher, which is pre-trained solely on image data and kept frozen. Specifically, intermediate feature maps before the segmentation head are refined using one of the three aforementioned attention strategies. This module enables the student to acquire refined visual knowledge distilled from the teacher.
**Semantic Knowledge Fusion Module.** Beyond visual supervision, we incorporate an auxiliary module to compensate for the lack of visual context during training. To improve semantic reasoning based on visual context, we adopt a fusion approach at the logit level of the student model to inject high-level semantic priors. Specifically, the generated linguistic descriptions are first transformed into text embeddings using a pre-trained vision-language text encoder, which aligns textual representations with the visual feature space [26]. The resulting text embeddings are represented as $e_{t}\in R^{c\times d}$, where *c* denotes the number of semantic classes and *d* the embedding dimension. We then employ a learnable projection layer to project the embedding dimension *d* to the class dimension *c*. The transformation is defined as:
  (5) $$z_{t}={\mathrm{Proj}}_{in}\left(e_{t}\right),$$
  where ${\mathrm{Proj}}_{in}$ denotes the learnable projection layer, and $z_{t}\in R^{c\times c}$ represents the projected text embeddings. This projection makes text representations compatible with the student model’s visual logits $z_{v}\in R^{HW\times c}$. Then, to integrate these modalities, we employ a cross-attention mechanism with $z_{v}$ as the query and $z_{t}$ as the key and value [45]. The semantic attention feature $z_{attn}\in R^{HW\times c}$ is computed as:
  (6) $$z_{attn}=\mathrm{softmax}\left(q\left(z_{v}\right)\cdot k{\left(z_{t}\right)}^{⊤}/\sqrt{c}\right)\cdot v\left(z_{t}\right),$$
  where *q*, *k*, and *v* denote the learnable linear projections for the query, key, and value, respectively. $z_{attn}$ captures the semantic inter-class relationships that the student needs to consider. Finally, $z_{attn}$ is projected back to the prediction space, producing the fused logits $z_{fused}\in R^{HW\times c}$. The resulting logits are combined with $z_{v}$ through a residual connection [46]:
  (7) $$\begin{array}{cc} \; & z_{fused}={\mathrm{Proj}}_{out}\left(z_{attn}\right),\\ \; & z=z_{fused}+z_{v}, \end{array}$$
  where ${\mathrm{Proj}}_{out}$ denotes the output projection layer, and $z\in R^{HW\times c}$ represents the final logits used for training. The final logits *z* are then used to compute the standard cross-entropy loss:
  (8) $$L_{CE}=\mathrm{CE}\left(\mathrm{softmax}\left(z\right),y_{gt}\right),$$
  where $y_{gt}$ denotes the ground-truth segmentation map.

The final training objective combines the cross-entropy loss $L_{CE}$ with the visual KD loss $L_{VisKD}$ defined in Equation (1):

(9) $$L_{total}=L_{CE}+\lambda L_{VisKD},$$

where λ is a hyperparameter that balances the contributions of the two loss terms.

**Inference Phase.** While the student model is trained with both visual and linguistic supervision, the teacher model and the semantic knowledge fusion module are discarded during the inference phase. Therefore, the original student model’s logits $z_{v}$ serve as the final output for inference. This design maintains the model’s computational efficiency and lightweight advantage while retaining the enhanced contextual knowledge acquired during training.

## 4. Experiments

### 4.1. Datasets

We evaluate our framework on public road scene datasets for semantic segmentation task, the Cambridge-driving labeled video (CamVid) [47] and Karlsruhe Institute of Technology and Toyota Technological Institute at Chicago (KITTI) [48]. CamVid consists of 701 images with pixel-level annotations across 11 classes, and KITTI consists of 445 images with 11 classes. We employ lower-resolution images than the original resolution in our experiments. Specifically, we use 384 × 288 for CamVid and 1280 × 384 for KITTI. For both datasets, we employ 2-fold cross-validation [49] for our experiments with a division of 350 and 351 images for CamVid, and 223 and 222 images for KITTI, respectively. Moreover, we allocate 10% of each fold as validation data during the training phase.

To reflect a limited FoV situation, we apply invalid black regions (i.e., zero-padding) at the edges of all input images. Specifically, for CamVid, invalid areas are set to 12.5% of both the image width and height. In the case of KITTI, the padding length is determined as 12.5% of the image height, and the same number of pixels is applied to the width, given that the width is significantly larger than the height. This limited FoV condition leads to incomplete visual information, simulating blind spots commonly encountered in real-world autonomous driving scenarios, where models are required to achieve robustness even under insufficient visual information.

### 4.2. Implementation Details

We employ three variants of the transformer-based semantic segmentation model SegFormer [50]: the larger-capacity B4 and B2 models as teachers and the lightweight B0 model as the student. The models contain 61.37 M, 24.73 M and 3.72 M parameters, respectively, corresponding to compression ratios of 6.06% (B4→B0) and 15.04% (B2→B0). For semantic guidance, we use the OpenCLIP ViT-B/16 text encoder pretrained with the LAION-2B laion2b_s34b_b88k checkpoint to extract text embeddings [51]. The effect of text encoder choice is further examined in Section 4.4.1.

All experimental settings are identical for both the teacher and student models. Specifically, we use the AdamW optimizer with a base learning rate of $6\times 10^{-5}$ following the optimizer settings in Xie et al. [50] and train the models for 35K iterations with a batch size of 4. Considering overfitting due to a small number of training samples, we apply random cropping with crop sizes of 288×288 for CamVid and 384×384 for KITTI. We also employ photometric distortion and random horizontal flipping with a 50% probability as data augmentation.

### 4.3. Joint Supervision from Attention-Based KD and Semantic Guidance

In this section, we evaluate the joint effect of attention-based KD and semantic guidance under limited FoV conditions. For clarity, the three attention module configurations are denoted as SAFD-C, SAFD-S, and SAFD-CS, corresponding to CAM, SAM, and CBAM (channel and spatial attention). We first compare the representative SAFD-CS configuration against several KD baselines to assess the benefits of attention-based KD and the additional effect of semantic guidance under limited FoV conditions. We then investigate the characteristics of semantic guidance across different attention-based distillation strategies and textual representations. Semantic segmentation performance is evaluated using two commonly adopted metrics: mean pixel accuracy (mPA) and mean intersection over union (mIoU). These complementary metrics provide a comprehensive and balanced evaluation across semantic classes, reducing the influence of class imbalance.

#### 4.3.1. Comparison with Representative KD Methods

We compare SAFD-CS with *Label* guidance against several representative KD baselines considering different categories of distillation, which include vanilla KD [9], AT [11], SP [35], RKD [36], ReviewKD [34], GDKD [52], and AttnFD [15]. For the CBAM-based distillation strategy, SAFD-CS without semantic guidance follows the AttnFD framework.

Table 2 summarizes the overall semantic segmentation performance using SegFormer-B2 and SegFormer-B4 as teachers on the CamVid and KITTI datasets. Most representative KD methods improve segmentation performance over the student model. Among them, an attention-based approach such as AttnFD consistently achieves stronger performance than conventional KD methods for both mPA and mIoU. By further incorporating semantic guidance, SAFD-CS (Label) achieves additional performance gains across different teacher architectures and datasets, consistently outperforming all compared KD baselines.

**Figure 2:**
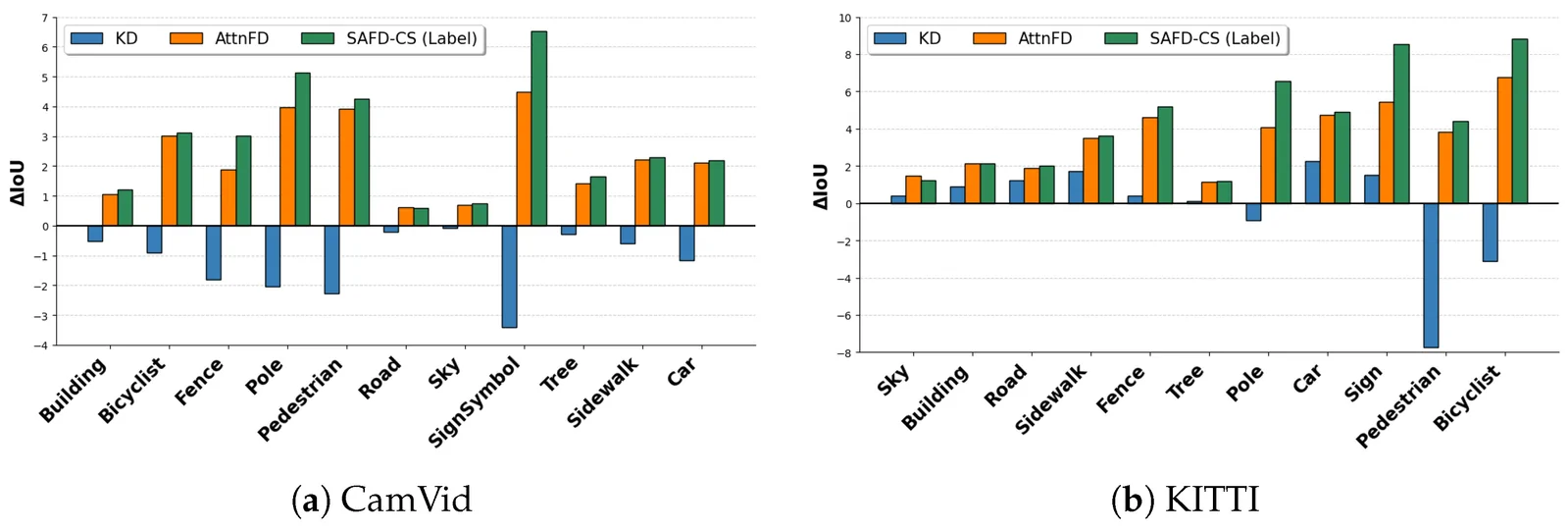
Per-class ΔIoU of different KD methods with the SegFormer-B2 teacher relative to the student model learned from scratch.

To further investigate the class-wise improvements, Figure 2 illustrates the per-class IoU improvement (ΔIoU) over the student model (learned from scratch) achieved by vanilla KD, AttnFD and SAFD-CS (Label) using a SegFormer-B2 teacher. On the CamVid dataset, vanilla KD exhibits performance degradation across all categories. In contrast, on the KITTI dataset, vanilla KD primarily improves dominant categories while noticeably degrading the performance of small object classes such as *pedestrian* and *bicyclist*. This overall degradation observed on CamVid is likely caused by the larger proportion of invalid padded regions compared to the KITTI dataset. Since vanilla KD directly matches the teacher predictions, the transferred supervision may contain noisy responses induced by these wide padded regions rather than meaningful semantic cues, ultimately leading to degraded performance. Despite these limitations, AttnFD substantially alleviates the degradation of challenging categories while maintaining competitive performance on dominant classes across both datasets by transferring more discriminative visual representations. Building upon this attention-based visual supervision, SAFD-CS (Label) further improves the recognition of challenging categories through semantic guidance, particularly for *bicyclist*, *pole*, *pedestrian*, and *signsymbol*.

Overall, these results indicate that attention-based KD transfers more discriminative visual knowledge than conventional KD methods under limited FoV conditions. Building upon this visual supervision, semantic guidance provides additional benefits, particularly for challenging object categories.

#### 4.3.2. Semantic Guidance with Attention-Based KD

To investigate the effects of different visual feature attention strategies and textual formats for semantic guidance, we compare three SAFD configurations with different textual representations.

Table 3 summarizes the overall mIoU results obtained using SegFormer-B2 and SegFormer-B4 teachers on the CamVid and KITTI datasets. On CamVid, incorporating semantic guidance enhances segmentation performance across all SAFD variants with the SegFormer-B2 teacher. However, although the stronger SegFormer-B4 teacher improves the student’s performance through KD, only marginal improvements or slight degradations are observed after incorporating semantic guidance. In contrast, a different trend is observed on KITTI. The performance without semantic guidance (w/o SG) remains similar for both teacher capacities, and semantic guidance consistently provides additional improvements for both teacher models except for a few combinations exhibiting minor degradation. Despite these dataset-specific observations, a common tendency emerges across both datasets. The additional improvements provided by semantic guidance are generally larger when visual distillation alone (w/o SG) yields relatively smaller improvements, whereas they become marginal as the effectiveness of visual distillation increases. This observation suggests that the impact of semantic guidance depends on how much improvement is already achieved through KD alone.

To further examine how semantic guidance interacts with attention-based visual distillation, we analyze the effects of different textual formats on each attention strategy. For SAFD-C, *Sentence* generally provides the largest improvements with the SegFormer-B2 teacher on both datasets, while *Keywords* also achieves larger gains than *Label*. This suggests that channel attention, which emphasizes global channel dependencies, may benefit more from semantically richer representations, such as *Keywords* and *Sentence*, than from coarse class-level descriptions provided by *Label*. However, this tendency is not consistently observed with the SegFormer-B4 teacher. On CamVid, all textual representations result in slight performance degradation, whereas *Keywords* achieves the best performance on KITTI. One possible explanation is that richer textual descriptions become less beneficial when the transferred visual representations may already capture sufficient semantic context, making additional contextual information redundant.

In contrast, SAFD-S exhibits a more consistent pattern across teacher capacities. *Label* consistently achieves the best performance on CamVid, whereas *Keywords* and *Sentence* show robust performance on KITTI. Since spatial attention primarily refines spatially discriminative features, explicit class identities provided by *Label* may be sufficient to alleviate spatial ambiguity under the more severely restricted FoV in CamVid. Meanwhile, KITTI preserves relatively richer spatial cues, suggesting that detailed semantic representations beyond explicit class identities may provide more effective guidance for spatial attention.

For SAFD-CS, *Label* generally yields competitive or superior performance across textual formats. As the attention mechanism jointly exploits both channel and spatial attention, the most effective textual representation appears to depend on how well it cooperates with both attention strategies. Consequently, concise and explicit semantic representations, such as *Label*, may provide more stable semantic guidance than richer textual descriptions, although the optimal textual format still varies depending on the dataset characteristics.

Overall, across the evaluated teacher and dataset settings, most configurations with semantic guidance outperform their corresponding configurations without semantic guidance, while every semantic-guided configuration remains superior to the student trained from scratch. The variation in performance gains and optimal textual formats across attention strategies indicates that the synergistic effects depend on how the semantic representation complements the transferred visual knowledge rather than on semantic richness alone. Taken together, these results show that semantic guidance and attention-based visual distillation can jointly enhance segmentation performance through configuration-dependent synergistic effects.

**Figure 3:**
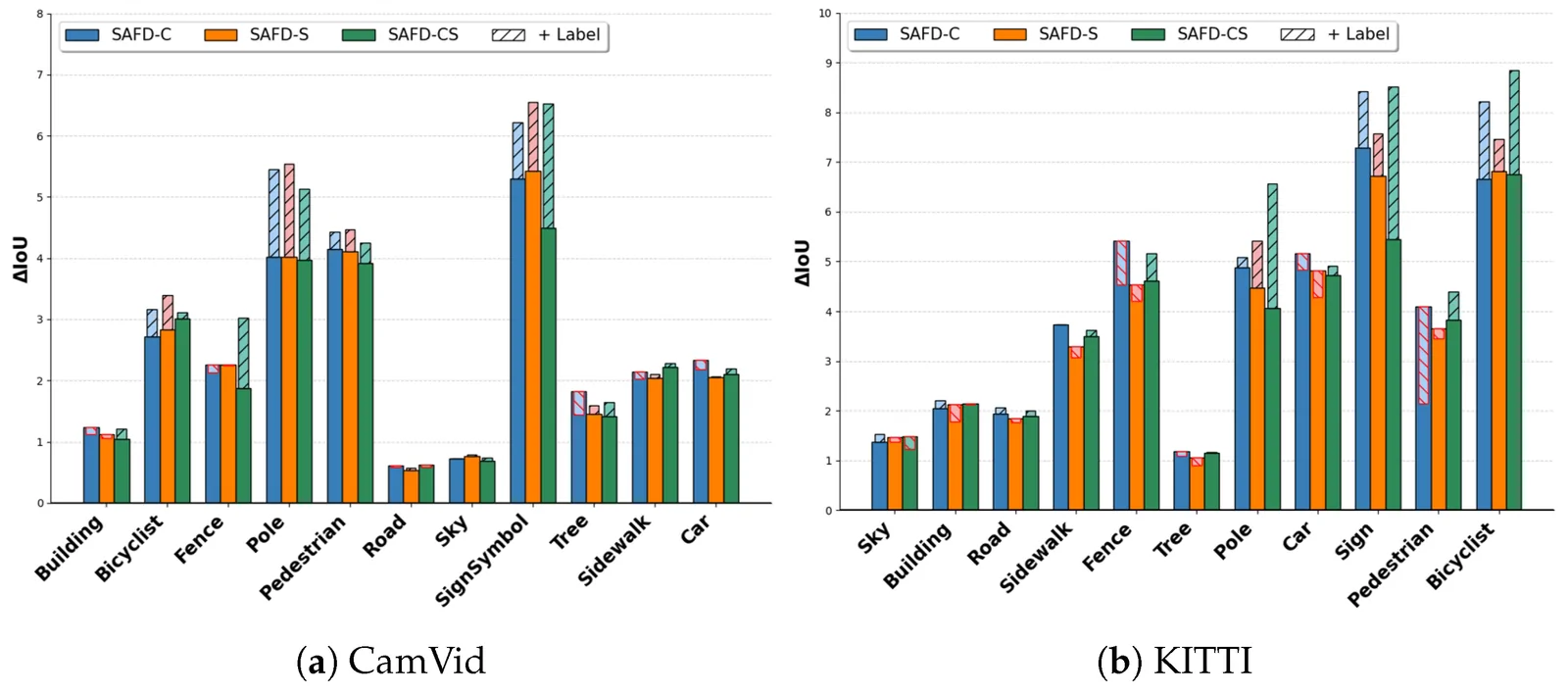
Per-class ΔIoU of different SAFD configurations using *Label* with the SegFormer-B2 teacher relative to the student learned from scratch. Solid bars denote the improvements from attention-based KD, whereas hatched bars represent the additional changes introduced by *Label* guidance. Red hatched bars indicate performance degradation.

Figure 3 further illustrates the per-class IoU improvement (ΔIoU) of each SAFD configuration using *Label* as semantic guidance with the SegFormer-B2 teacher relative to the student trained from scratch. Across both datasets, semantic guidance tends to provide larger additional performance gains for categories that benefit less from distillation without semantic guidance. This tendency is particularly evident for challenging categories such as *bicyclist*, *pole*, and *signsymbol*, which are particularly important in autonomous driving, as they frequently appear and require precise recognition within a limited FoV. Among the SAFD variants, SAFD-CS achieves relatively smaller gains from visual distillation alone on the *signsymbol* class across both datasets. However, after incorporating semantic guidance, it obtains the largest additional improvements, resulting in competitive or even superior final performance compared with the other attention-based KD strategies. Meanwhile, some cases, such as SAFD-C (Label) on KITTI, exhibit degradations in several classes, which is consistent with their relatively limited improvements in Table 3.

Taken together, these class-wise observations are consistent with the previous results, indicating that semantic guidance is particularly beneficial when semantic supervision provided by visual distillation alone remains limited.

### 4.4. Ablation and Additional Analysis

We provide further analysis through ablation studies, qualitative evaluations, and generalizability assessments. First, we conduct ablation studies on the choice of text encoder and the sensitivity of the training hyperparameter. Second, we present qualitative results to better understand the sources of performance improvements. Finally, we examine the generalizability of our framework by analyzing its performance under limited training data and the reliability of its predictive probabilities.

#### 4.4.1. Choice of Text Encoder

To assess the effect of text encoder choice, we compare several pretrained text encoder configurations from OpenCLIP [51], CLIP [26], SigLIP [53], and BERT [54]. All experiments are conducted using SAFD-CS (Label) with the SegFormer-B2 teacher on both CamVid and KITTI. The *Label* representation and all other training settings are kept fixed across the encoders.

As shown in Table 4, all evaluated text encoder configurations outperform both the standalone student and SAFD-CS (w/o SG) on both datasets, indicating that the performance benefit of semantic guidance is not restricted to a specific text encoder. Among them, OpenCLIP ViT-B/16 pretrained on LAION-2B achieves the highest mIoU on KITTI and the joint-highest mIoU on CamVid, providing the strongest overall performance across the two datasets. These results support the suitability of the text encoder used in the main experiments while demonstrating that SAFD can utilize semantic representations obtained from different pretrained text encoders.

#### 4.4.2. Hyperparameter Sensitivity

**Figure 4:**
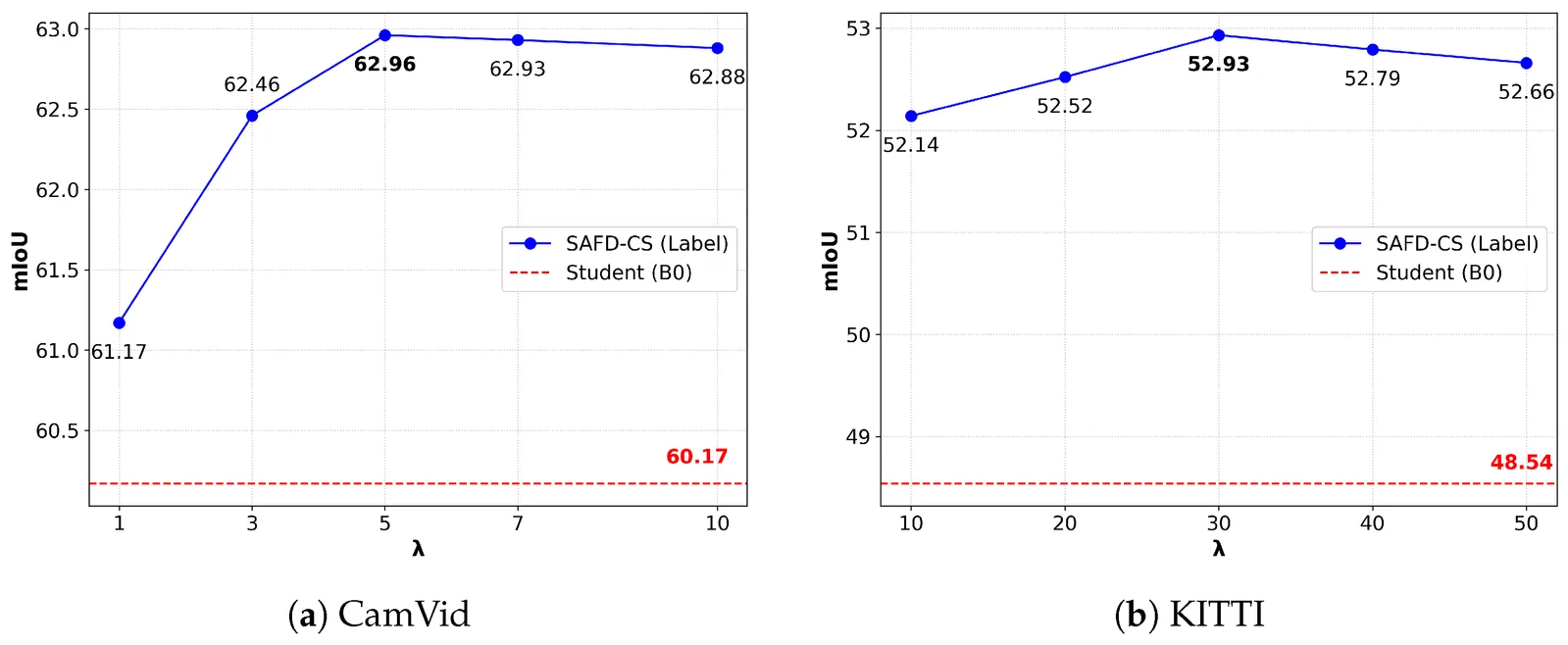
Sensitivity analysis of the KD loss weight λ for SAFD-CS (Label) on the CamVid and KITTI datasets.

We analyze the sensitivity of the hyperparameter λ, which controls the contribution of the visual KD loss. Figure 4 illustrates the mIoU obtained with different λ values under the SAFD-CS (Label) setting with the SegFormer-B2 teacher on the CamVid and KITTI datasets. On both datasets, increasing λ consistently improves performance up to an intermediate value, indicating that sufficient visual KD supervision allows the student to better leverage semantic knowledge during training. Specifically, the best performance is achieved at λ=5 on CamVid and λ=30 on KITTI. Beyond the optimum, only marginal changes are observed despite further increasing λ, suggesting that the benefit of semantic guidance becomes relatively insensitive to λ once sufficient visual supervision is provided. This stability is particularly evident on CamVid, where the performance remains nearly unchanged over a broad range of λ values.

#### 4.4.3. Visualization

**Figure 5:**
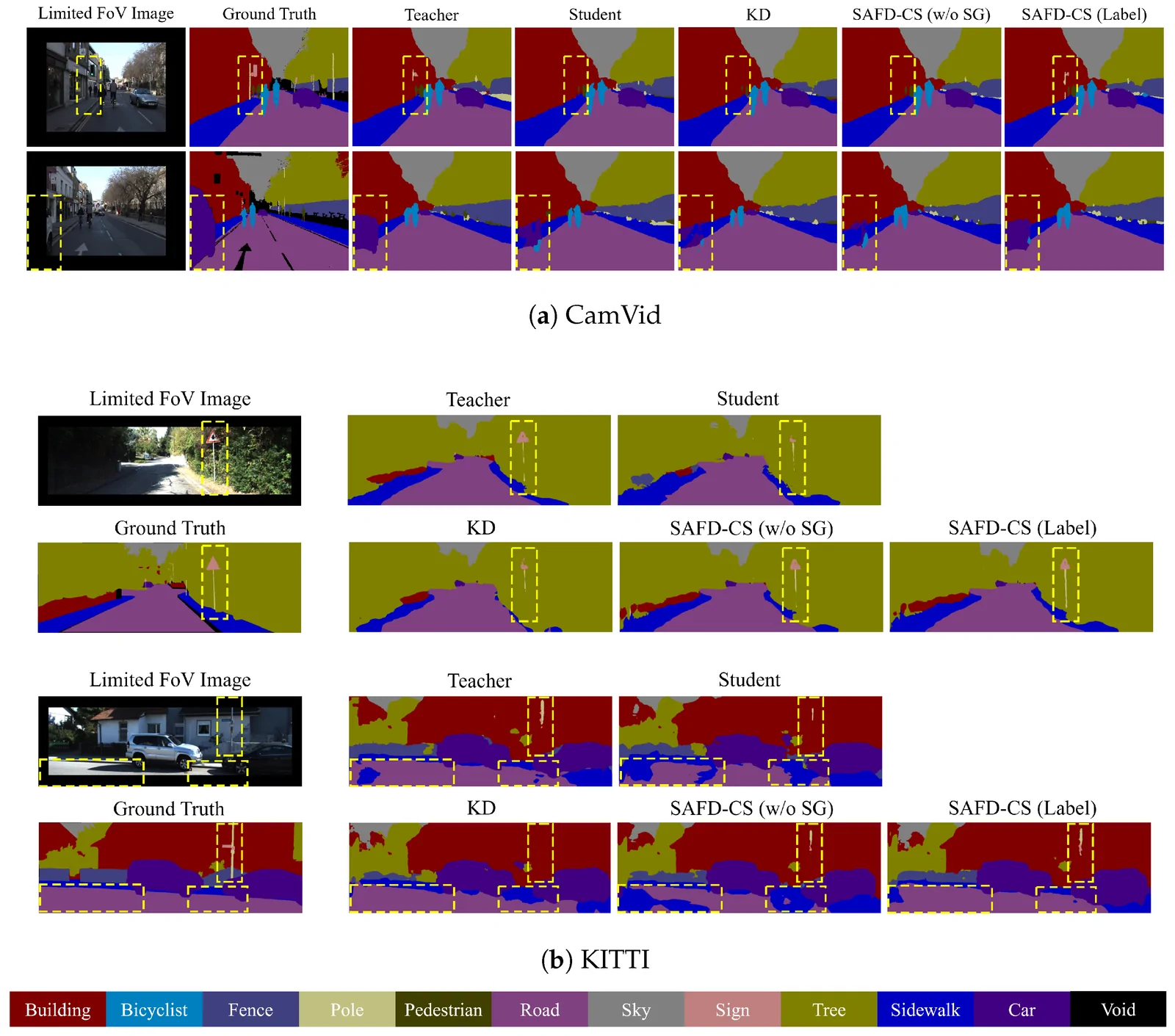
Visualization for semantic segmentation results on the CamVid and KITTI datasets.

**Segmentation Result.** Figure 5 presents qualitative semantic segmentation results on the CamVid and KITTI datasets. SAFD-CS is adopted as the representative attention-based distillation strategy, while the *Label* representation is used to evaluate the effect of semantic guidance.

As shown in Figure 5, on both datasets, vanilla KD captures larger categories (e.g., *building*, *tree*, and *road*) well, but it struggles to recognize smaller objects (e.g., *pole* and *sign*), and their predicted boundaries are often noisy. In contrast, SAFD-CS without semantic guidance (SAFD-CS (w/o SG)) improves the recognition of smaller objects compared with vanilla KD while preserving the performance of dominant categories. However, distinguishing visually challenging regions under limited FoV conditions remains difficult. Crucially, SAFD-CS (Label) further improves the recognition of small-scale instances while maintaining the prediction quality for larger categories.

Furthermore, the cluttered and noisy predictions around the padded regions observed in SAFD-CS (w/o SG) are noticeably mitigated. Specifically, in Figure 5a, both vanilla KD and SAFD-CS (w/o SG) produce only sparse and fragmented predictions for the *car* in the restricted area, whereas SAFD-CS (Label) predicts a broader and more coherent *car* region. In Figure 5b, while vanilla KD exhibits a bias toward dominant large-scale categories such as *road*, resulting in smooth predictions in the padded regions, it fails to recognize small objects such as *pole* and *signsymbol*. In contrast, SAFD-CS (Label) maintains comparable prediction quality for large regions while better recognizing small objects.

These qualitative results are consistent with the quantitative improvements reported in Section 4.3.1 and Section 4.3.2, demonstrating that semantic guidance not only improves the recognition of visually challenging categories while maintaining the overall prediction quality for dominant classes but also compensates for limited visual context.

**Figure 6:**
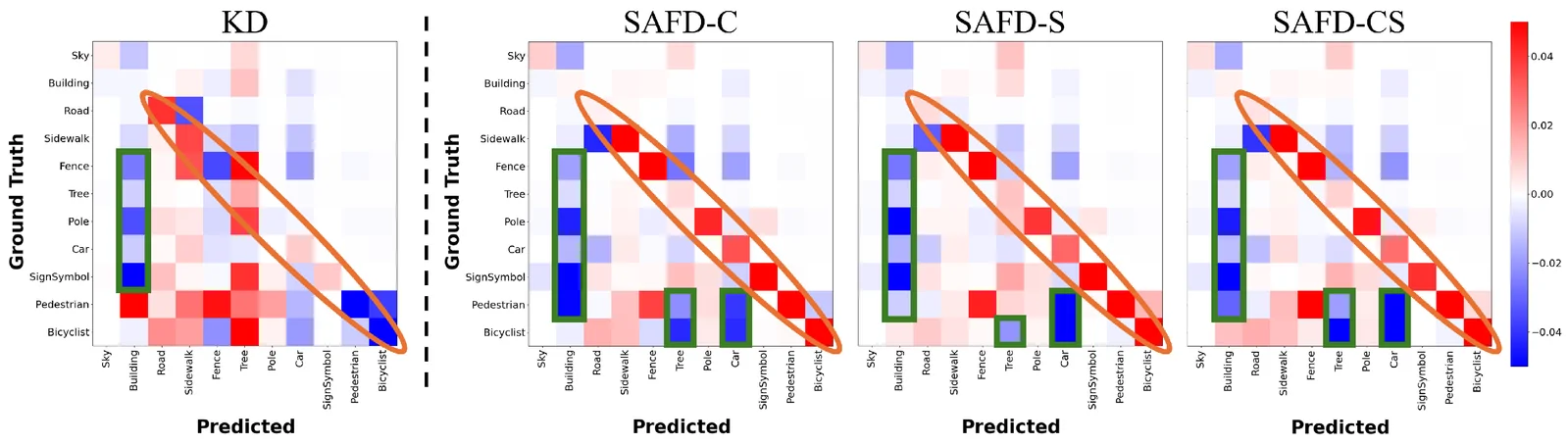
Confusion matrix difference maps of different KD methods relative to the student model on the KITTI dataset. Each map is computed by subtracting the normalized confusion matrix of the student model from that of the compared method ($CM_{\mathrm{KD}}-CM_{\mathrm{student}}$). Red and blue denote positive and negative differences, respectively. Red values on the diagonal indicate improved correct classifications, whereas blue values on the off-diagonal indicate reduced inter-class confusions relative to the student model. Orange circles indicate the diagonal cells and green boxes highlight representative examples of improvements.

**Figure 7:**
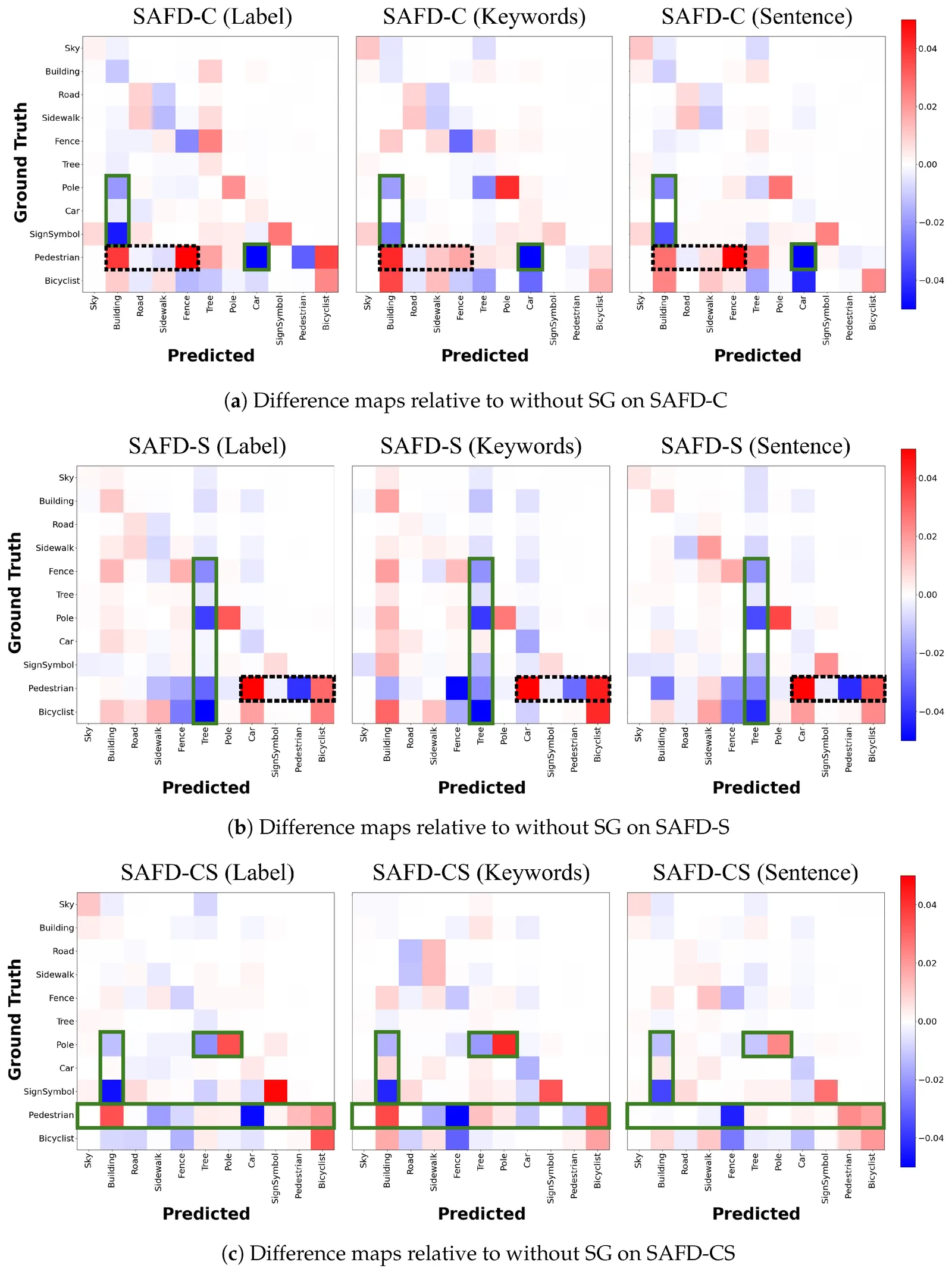
Confusion matrix difference maps of semantic guidance with different textual representations relative to the corresponding KD method without semantic guidance (w/o SG) on the KITTI dataset. Each map is computed by subtracting the normalized confusion matrix of each attention-based KD method without semantic guidance (w/o SG) from that of the semantic-guided (w/ SG) method ($CM_{w/\;\mathrm{SG}}-CM_{w/o\;\mathrm{SG}}$). Green boxes highlight representative examples of improvements, whereas black dotted boxes indicate representative degradation patterns.

**Confusion Matrix Difference.** While the segmentation results demonstrate spatial improvements, we further analyze the class-wise prediction behavior and inter-class confusions through confusion matrix difference maps shown in Figure 6 and Figure 7. Pixel-level confusion matrices are computed from the predicted and ground-truth labels over all pixels in the test set. Each confusion matrix is row-wise normalized to account for class imbalance, and the difference map is obtained by subtracting the normalized confusion matrix of the baseline method from that of the compared method.

In Figure 6, we show the confusion matrix difference maps ($CM_{\mathrm{KD}}-CM_{\mathrm{student}}$), where KD includes vanilla KD [9] and SAFD variants without semantic guidance (SAFD w/o SG), to analyze the effectiveness of visual attention in distillation. Compared to vanilla KD, the SAFD variants exhibit stronger red values on the diagonal cells and blue values on the off-diagonal cells. In particular, as highlighted by the orange circles along the diagonal cells, a pronounced increase in correct classification is observed for small objects (e.g., *signsymbol*, *pedestrian* and *bicyclist*). Meanwhile, the green boxes on the off-diagonal cells highlight a notable reduction in misclassifications of small objects (e.g., *pole*, *signsymbol*, *pedestrian* and *bicyclist*) into large categories (e.g., *building*, *tree*, and *car*). Although these improvements appear in both SAFD-C and SAFD-S, SAFD-C exhibits more robust predictions for these small categories. Furthermore, SAFD-CS integrates the characteristics of both mechanisms, thereby exhibiting the combined behavior. As a result, these observations suggest that SAFD produces more discriminative class-wise predictions than vanilla KD by improving the recognition of small foreground objects while enhancing their separation from large background categories.

In Figure 7, we investigate how semantic guidance further modifies these class-wise prediction patterns for each attention-based distillation strategy, showing confusion matrix difference maps ($CM_{w/\;\mathrm{SG}}-CM_{w/o\;\mathrm{SG}}$). Figure 7a–c illustrates the difference maps introduced by semantic guidance in the corresponding SAFD-C, SAFD-S, and SAFD-CS configurations, respectively. Although different textual representations are adopted, each attention-based distillation strategy shows a similar tendency and a consistent confusion pattern.

We explain the details of each strategy with highlights in this figure. As indicated with green boxes under SAFD-C in Figure 7a, misclassifications of *pole* and *signsymbol* as *building*, along with *pedestrian* as *car*, are substantially reduced. Conversely, errors where *pedestrian* is mistaken for *building* and *fence* increase, as highlighted by the black dotted boxes. These observations suggest that semantic guidance redistributes prediction errors according to the characteristics of classes through channel attention. In particular, semantic guidance effectively reinforces channel-wise semantic discrimination for semantically distinct categories, such as the *building*–*pole*, *building*–*signsymbol*, and *pedestrian*–*car* confusion pairs. In contrast, ambiguities involving *pedestrian* and surrounding structures (e.g., *building* and *fence*) become more pronounced, where adjacent object boundaries make accurate localization more challenging. This learning scheme aligns with the characteristics of channel attention, which primarily emphasizes channel-wise feature discrimination rather than detailed spatial reasoning. Therefore, while semantic guidance introduces additional class-specific semantic priors, the resulting prediction changes remain primarily shaped by the channel-attention strategy.

For SAFD-S in Figure 7b, green boxes highlight that semantic guidance substantially reduces the misclassification of foreground objects, including *pole*, *pedestrian*, and *bicyclist*, as *tree*. Notably, the most pronounced improvement is observed for the *pole*–*tree* confusion pair. Although *pole* and *tree* share similar vertical visual patterns, the additional semantic priors reinforce their distinct semantic identities, resulting in more reliable discrimination. In contrast, as highlighted by the black dotted boxes, confusions among foreground objects increase, particularly when *pedestrian* is misclassified as *car* or *bicyclist*. These observations indicate that semantic guidance is preferentially exploited together with the spatial representations emphasized by spatial attention, thereby improving foreground–background separation. However, the same semantic priors appear insufficient for ambiguities among foreground object categories, which rely more heavily on channel-wise semantic discrimination than spatial representations. Consequently, although semantic guidance consistently provides additional class-specific semantic priors, the resulting prediction changes remain primarily shaped by the spatial-attention strategy.

The results of SAFD-CS exhibit the combined behaviors observed in both SAFD-C and SAFD-S. As highlighted by the green boxes in Figure 7c, SAFD-CS consistently produces more stable predictions for *pedestrian* across all textual representations than either SAFD-C or SAFD-S. This improvement is achieved by simultaneously alleviating the degradation patterns observed in the individual channel- and spatial-attention strategies while preserving their representative strengths. This observation reflects the sequential integration of CAM and SAM in CBAM, enabling semantic priors to be jointly exploited through both channel-wise semantic discrimination and spatial representations.

In summary, semantic guidance produces distinct class-wise prediction changes depending on the adopted attention-based KD strategy, thereby redistributing prediction errors in a strategy-specific manner. Although minor variations exist across different textual representations, the overall confusion patterns are primarily governed by the chosen KD strategy. These findings demonstrate that semantic guidance consistently provides class-specific semantic priors, while their mode of utilization varies across different attention mechanisms.

#### 4.4.4. Generalizability Analysis of SAFD

We assess the generalizability of SAFD from two complementary perspectives: the preservation of segmentation performance as the amount of training data decreases and the reliability and generalizability of its predictive probabilities on unseen test samples.

**Generalization under Limited Training Data.** To examine the generalization behavior of the framework under limited training data, we vary the proportion of training samples used to train the student to 100%, 50%, and 30% while leaving the validation and test sets unchanged. At each proportion, we compare SAFD-CS (Label) with a standalone student trained using the same subset. The reduced training subsets are nested within the full training set. All other training settings—including data augmentation, the optimizer, batch size, and total training iterations—are kept identical to those described in Section 4.2. Since the number of training iterations is fixed, samples in the reduced subsets are revisited more frequently, providing increasingly challenging conditions with a higher risk of overfitting.

Table 5 reports the training and validation segmentation losses, the corresponding train–validation loss gaps, and the test mIoU for SAFD-CS (Label) using the SegFormer-B2 teacher. As the training proportion decreases, the training loss declines while the validation loss increases, resulting in a wider loss gap and indicating a greater tendency to fit the reduced subsets. Despite this more challenging condition, the improvement over the standalone student grows on both CamVid and KITTI datasets. This growing margin suggests that the generalization benefit of SAFD is not diminished by the reduction in training samples and that the framework better preserves its test performance than the student under limited training data.

**Figure 8:**
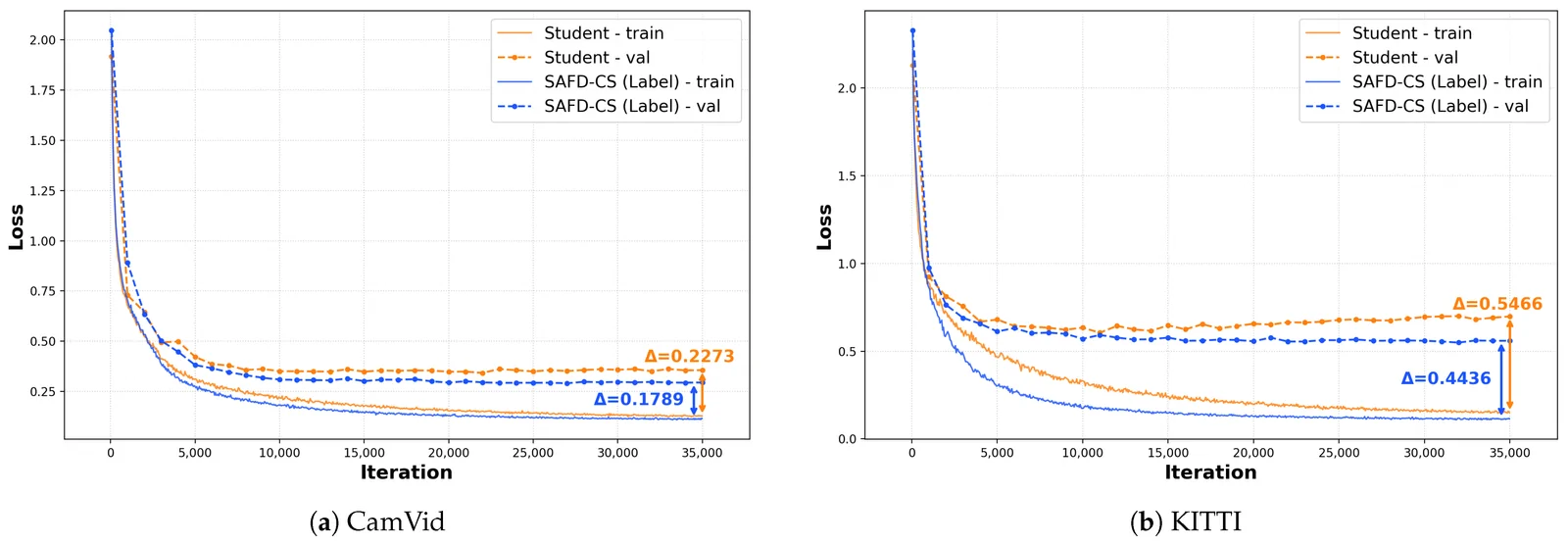
Training and validation loss curves of the standalone student and SAFD-CS (Label) trained using 30% of the training samples on the CamVid and KITTI datasets. Solid and dashed lines represent the training and validation losses, respectively. The annotated arrows and Δ values indicate the differences between the corresponding training and validation curves at the final training iteration.

The loss curves in Figure 8 further clarify this behavior under the most limited setting by comparing the training and validation segmentation losses of the standalone student and SAFD-CS (Label) using 30% of the training samples. On CamVid, the validation losses of both models stabilize as training proceeds, while SAFD-CS (Label) maintains a lower validation loss and a smaller endpoint separation between the training and validation curves. The difference is more pronounced on KITTI. Although the training loss of the standalone student continues to decrease, its validation loss gradually increases after the initial convergence. In contrast, SAFD-CS (Label) maintains a substantially lower and more stable validation loss during the later training stages.

Together, the test results and loss curves show that SAFD-CS (Label) better preserves segmentation performance and exhibits more stable generalization than the standalone student as the amount of training data decreases. These results suggest that the additional visual and semantic supervision helps mitigate the effects of overfitting to the reduced training subsets, thereby limiting performance degradation on unseen samples.

**Reliability and Generalizability.** Beyond evaluating performance under limited training data, we further examine whether the predictive probabilities produced by SAFD reliably reflect prediction correctness on unseen test samples. We first visualize the calibration behavior of representative semantic guidance configurations and then quantitatively evaluate all textual representations on the KITTI dataset.

**Figure 9:**
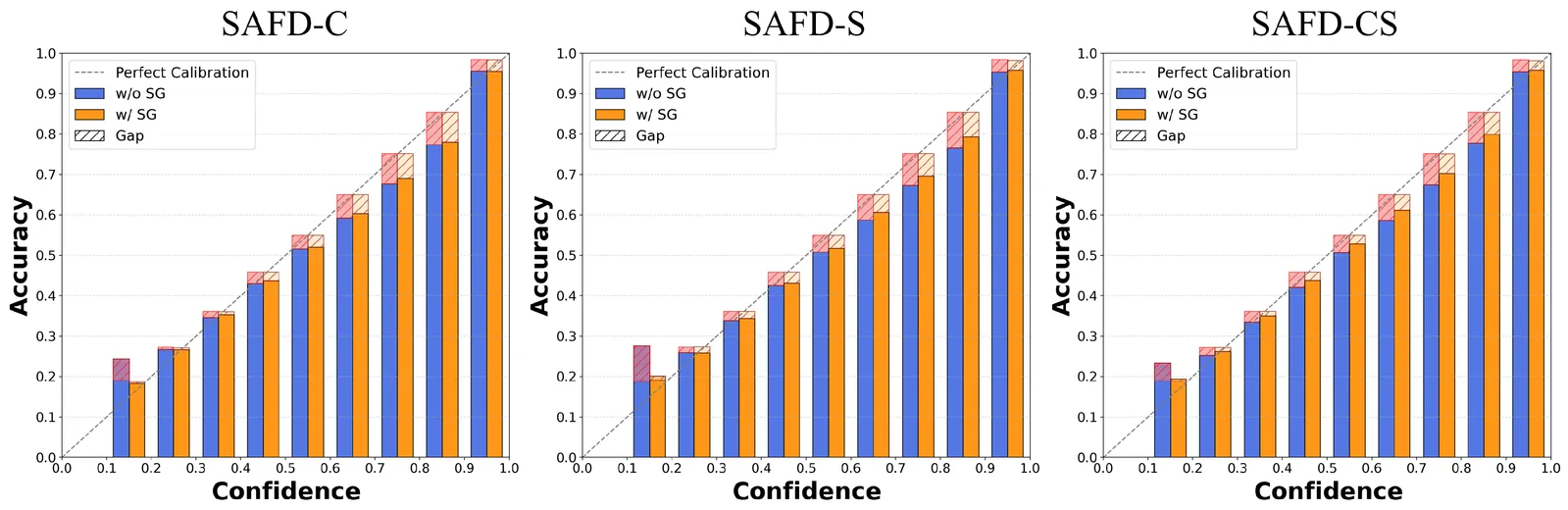
Reliability diagrams of each SAFD variant with and without its semantic guidance (SG) configuration achieving the highest mIoU on the KITTI dataset. For each SAFD configuration, the selected textual representations are *Sentence* for SAFD-C, *Keywords* for SAFD-S, and *Label* for SAFD-CS.

Figure 9 presents the reliability diagrams [55], which visualize the agreement between prediction confidence and empirical accuracy. A calibrated model follows the diagonal line, while deviations from the diagonal indicate overconfidence or underconfidence. Therefore, bars closer to the diagonal indicate that the reported confidence more accurately reflects the observed probability of a correct prediction. Semantic guidance (w/ SG) consistently improves confidence calibration across all SAFD variants shown in the figure. In particular, predictions with low confidence (0.1–0.2), which are noticeably miscalibrated in the models without semantic guidance (w/o SG), become substantially better aligned with their empirical accuracy after incorporating semantic guidance. Similar improvements are also observed across the remaining confidence intervals.

Additionally, we quantitatively evaluate prediction reliability using the Expected Calibration Error (ECE) [56] and Negative Log-Likelihood (NLL) [55]. ECE measures the overall discrepancy between prediction confidence and empirical accuracy, whereas NLL evaluates the probability assigned to the ground-truth labels and strongly penalizes confident incorrect predictions. Therefore, ECE and NLL provide complementary assessments of whether the predictive probabilities meaningfully reflect prediction correctness. For semantic segmentation, ECE is computed at the pixel level by treating each pixel as an individual prediction:

(10) $$\mathrm{ECE}=\sum_{m=1}^{M}\frac{|B_{m}|}{N}\left|\mathrm{acc}\left(B_{m}\right)-\mathrm{conf}\left(B_{m}\right)\right|,$$

where $B_{m}$ denotes the set of pixels whose confidence falls into the *m*-th confidence bin, *N* is the total number of pixels in the evaluation set, $\mathrm{acc}(B_{m})$ is the average pixel accuracy within the bin, and $\mathrm{conf}(B_{m})$ is the average confidence. The pixel-wise NLL is also calculated as:

(11) $$\mathrm{NLL}=-\frac{1}{N}\sum_{i=1}^{N}\log p_{i}\left(y_{i}\right),$$

where $y_{i}$ denotes the ground-truth class of the *i*-th pixel and $p_{i}\left(y_{i}\right)$ is the predicted probability assigned to that class.

As shown in Table 6, all SAFD configurations achieve lower ECE and NLL than the standalone student. Compared with their corresponding configurations without semantic guidance, most configurations with semantic guidance further reduce ECE, indicating better agreement between prediction confidence and empirical accuracy. These trends are broadly consistent with the mIoU results in Section 4.3.2 despite minor differences in the optimal textual representation. Although the NLL results are more dependent on the combination of attention strategy and textual representation, each SAFD variant has a textual format that jointly achieves its lowest ECE and NLL. This lower NLL indicates that these configurations assign higher likelihood to the ground-truth labels of unseen test samples, suggesting that the learned predictive distributions generalize more effectively beyond the training data. Together with the reduced ECE, these results show that semantic guidance can improve not only segmentation performance but also prediction reliability, thereby supporting the generalizability of SAFD from a likelihood-based perspective.

## 5. Discussion

The experimental results suggest that semantic guidance does not simply provide additional supervision but rather serves as complementary semantic supervision when visual distillation alone is insufficient. As observed in our experiments, the contribution of semantic guidance becomes more pronounced when the visual knowledge transferred through KD remains limited. This behavior indicates that linguistic semantic supervision compensates for semantic information that has not been effectively captured from visual supervision.

Another notable observation is that no single textual representation consistently achieves the best performance across different attention-based distillation strategies or datasets. Instead, the optimal textual representation varies according to the characteristics of the transferred visual representations and the target dataset. This suggests that richer semantic information does not necessarily lead to more effective semantic guidance; rather, the interaction between linguistic and visual representations plays a critical role in determining its effectiveness.

Beyond these findings, an interesting future direction arises from a structural perspective. SAFD incorporates semantic information only in the prediction space through logit-level fusion. While this design preserves the original visual representations transferred from the teacher and avoids additional feature-level optimization, it does not enable direct interaction between visual and semantic representations during feature learning. In future work, we will investigate the effectiveness of this feature-level integration and analyze the interaction between the two modalities.

## 6. Conclusions

This paper investigated the effectiveness of incorporating linguistic semantic knowledge with visual attention in KD for semantic segmentation under limited FoV conditions in driving scenes. To this end, we designed a framework named SAFD, which combines visual attention-based feature distillation and semantic guidance derived from LLMs without introducing additional inference cost. SAFD supports various attention-based distillation strategies and different types of text prompts, enabling a systematic analysis of how semantic guidance interacts with different forms of visual supervision.

Experiments demonstrated that semantic guidance generally improves attention-based visual distillation under limited FoV conditions and is particularly beneficial for visually challenging categories. Our analyses revealed that semantic guidance provides class-specific semantic priors that are exploited differently across attention mechanisms. Moreover, the benefits of semantic guidance are not tied to a particular text encoder, and it improves generalization by producing predictive probabilities that more faithfully reflect prediction correctness on unseen samples.

Overall, these findings suggest that linguistic semantic knowledge can effectively complement visual supervision for improved KD. We believe that this work provides useful insights into integrating language priors with visual knowledge transfer and encourages further exploration of vision-language collaboration for semantic segmentation and other dense prediction tasks.

## Figures and Tables

**Table 1:** Generated descriptions by GPT-5 in *Keywords* and *Sentence* formats.

Class	Keywords (k=5)
Sky	Sky, Atmosphere, Horizon, Clouds, Airspace
Building	Building, Architecture, Facade, Structure, Windows
Road	Road, Asphalt, Lane, Driveway, Trafficway
Sidewalk	Sidewalk, Curb, Walkway, Pedway, Paving
Fence	Fence, Boundary, Pickets, Barrier, Perimeter
Tree	Tree, Foliage, Trunk, Branches, Canopy
Pole	Pole, Slender, Vertical, Cable, Metallic
Car	Car, Vehicle, Windshield, Engine, Parking
SignSymbol	SignSymbol, Traffic sign, Pictogram, Instruction, Regulation
Pedestrian	Pedestrian, Crosswalk, Footsteps, Walker, Striding
Bicyclist	Bicyclist, Helmet, Pedals, Wheels, Cycling
**Class**	**Sentence**
Sky	The area above the earth, usually blue during the day with clouds.
Building	A tall structure made of concrete and glass that houses offices and apartments.
Road	A paved surface for vehicles to drive on, often marked with lanes.
Sidewalk	A paved path beside the road for people to walk safely.
Fence	A wooden or metal barrier that encloses a yard or property.
Tree	A tall plant with a trunk, branches, and leaves providing shade.
Pole	A vertical post used to support electrical wires or street lamps.
Car	A motor vehicle with four wheels used for transportation.
SignSymbol	A traffic sign or symbol used to guide or warn drivers and pedestrians.
Pedestrian	A person walking on the sidewalk or crossing the street.
Bicyclist	A person wearing a helmet riding a bicycle on the street.

**Table 2:** Comparison of semantic segmentation performance among several KD methods on the CamVid and KITTI datasets. Results are presented as mean ± standard deviation over three random seeds and the highest score for each dataset and teacher is highlighted in bold.

		T: SegFormer-B2		T: SegFormer-B4	
DB	Method	mPA	mIoU	mPA	mIoU
CamVid	Teacher	72.85	65.47	73.62	66.52
	Student	67.79	60.17	67.79	60.17
	KD [9]	66.52 ± 0.11	58.96 ± 0.17	67.95 ± 0.20	60.28 ± 0.17
	AT [11]	68.05 ± 0.09	60.42 ± 0.14	67.97 ± 0.16	60.49 ± 0.12
	SP [35]	67.97 ± 0.09	60.28 ± 0.17	67.84 ± 0.09	60.30 ± 0.09
	RKD [36]	69.19 ± 0.13	61.66 ± 0.12	68.93 ± 0.13	61.40 ± 0.17
	ReviewKD [34]	68.41 ± 0.26	60.91 ± 0.24	68.47 ± 0.08	60.93 ± 0.13
	GDKD [52]	68.05 ± 0.05	60.28 ± 0.16	67.84 ± 0.16	60.21 ± 0.17
	AttnFD [15]	69.72 ± 0.09	62.47 ± 0.11	70.71 ± 0.03	63.38 ± 0.06
	SAFD-CS (Label)	**70.46 ± 0.17**	**62.96 ± 0.10**	**70.72 ± 0.04**	**63.44 ± 0.18**
KITTI	Teacher	61.93	54.82	64.01	56.91
	Student	55.97	48.54	55.97	48.54
	KD [9]	54.94 ± 0.16	48.23 ± 0.10	56.94 ± 0.40	49.12 ± 0.37
	AT [11]	56.51 ± 0.49	48.87 ± 0.40	56.80 ± 0.24	49.10 ± 0.26
	SP [35]	56.67 ± 0.42	48.95 ± 0.37	56.61 ± 0.44	48.91 ± 0.43
	RKD [36]	59.38 ± 0.50	51.31 ± 0.33	58.77 ± 0.46	50.97 ± 0.41
	ReviewKD [34]	57.29 ± 0.32	49.60 ± 0.29	57.33 ± 0.07	49.63 ± 0.15
	GDKD [52]	56.60 ± 0.33	48.94 ± 0.35	56.71 ± 0.36	49.01 ± 0.35
	AttnFD [15]	59.59 ± 0.14	52.13 ± 0.08	59.88 ± 0.37	52.18 ± 0.26
	SAFD-CS (Label)	**61.03 ± 0.14**	**52.93 ± 0.13**	**60.40 ± 0.29**	**52.50 ± 0.16**

**Table 3:** Comparison of semantic segmentation performance (mIoU) across different attention-based refinement strategies and textual formats on the CamVid and KITTI datasets. Values in parentheses indicate the changes over the corresponding SAFD configuration without semantic guidance (w/o SG), with performance improvements shown in red and degradations in blue. The highest performance within each attention strategy and teacher model is highlighted in bold. SG denotes semantic guidance.

			T: SegFormer-B2	T: SegFormer-B4
DB	Method	Format	mIoU	mIoU
CamVid	SAFD-C	w/o SG	62.65 ± 0.10	**63.46 ± 0.11**
		Label	62.85 ± 0.09 (+0.20)	63.42 ± 0.11 (−0.04)
		Keywords	62.90 ± 0.05 (+0.25)	63.42 ± 0.03 (−0.04)
		Sentence	**62.95 ± 0.07** (+0.30)	63.33 ± 0.17 (−0.13)
	SAFD-S	w/o SG	62.59 ± 0.19	63.42 ± 0.02
		Label	**62.93 ± 0.14** (+0.34)	**63.53 ± 0.29** (+0.11)
		Keywords	62.85 ± 0.21 (+0.26)	63.44 ± 0.08 (+0.02)
		Sentence	62.74 ± 0.13 (+0.15)	63.47 ± 0.21 (+0.05)
	SAFD-CS	w/o SG	62.47 ± 0.11	63.38 ± 0.06
		Label	**62.96 ± 0.10** (+0.49)	63.44 ± 0.18 (+0.06)
		Keywords	62.87 ± 0.13 (+0.40)	**63.45 ± 0.16** (+0.07)
		Sentence	62.69 ± 0.13 (+0.22)	63.33 ± 0.24 (−0.05)
KITTI	SAFD-C	w/o SG	52.51 ± 0.09	52.45 ± 0.33
		Label	52.52 ± 0.21 (+0.01)	52.41 ± 0.16 (−0.04)
		Keywords	52.58 ± 0.25 (+0.07)	**53.09 ± 0.31** (+0.64)
		Sentence	**52.63 ± 0.40** (+0.12)	52.72 ± 0.53 (+0.27)
	SAFD-S	w/o SG	52.24 ± 0.13	52.09 ± 0.18
		Label	52.29 ± 0.36 (+0.05)	52.28 ± 0.22 (+0.19)
		Keywords	**52.62 ± 0.13** (+0.38)	**52.84 ± 0.00** (+0.75)
		Sentence	52.58 ± 0.27 (+0.34)	52.79 ± 0.16 (+0.70)
	SAFD-CS	w/o SG	52.13 ± 0.08	52.18 ± 0.26
		Label	**52.93 ± 0.13** (+0.80)	**52.50 ± 0.16** (+0.32)
		Keywords	52.47 ± 0.23 (+0.34)	52.02 ± 0.17 (−0.16)
		Sentence	52.54 ± 0.06 (+0.41)	52.37 ± 0.22 (+0.19)

**Table 4:** Comparison of semantic segmentation performance (mIoU) across different text encoders for SAFD-CS (Label) on the CamVid and KITTI datasets. Bold values indicate the best result per dataset.

Method	Text Encoder	Configuration	Pretraining	CamVid	KITTI
Student		-	-	60.17	48.54
SAFD-CS	(w/o SG)	-	-	62.47 ± 0.11	52.13 ± 0.08
	OpenCLIP [51]	ViT-B/16	LAION-2B	**62.96 ± 0.10**	**52.93 ± 0.13**
		ViT-B/32	LAION-2B	62.75 ± 0.07	52.57 ± 0.03
	CLIP [26]	ViT-B/16	OpenAI	62.91 ± 0.26	52.66 ± 0.03
		ViT-B/32	OpenAI	**62.96 ± 0.06**	52.71 ± 0.08
	SigLIP [53]	ViT-B/16	WebLI	62.92 ± 0.21	52.69 ± 0.14
	BERT [54]	Base (uncased)	BooksCorpus + Wikipedia	62.79 ± 0.16	52.50 ± 0.11

**Table 5:** Generalization performance of SAFD-CS (Label) under different training data proportions on the CamVid and KITTI datasets. Values in parentheses denote the mIoU improvements over the standalone student trained using the corresponding training data proportion, and bold values indicate the best performance per dataset.

DB	Proportion	Train Loss $L_{\mathrm{train}}$	Validation Loss $L_{\mathrm{val}}$↓	Train–Val Loss Gap ($L_{\mathrm{val}}-L_{\mathrm{train}}$)↓	Test mIoU (%) ↑
CamVid	100%	0.1489	**0.2338**	**0.0849**	**62.96** (+2.79)
	50%	0.1305	0.2596	0.1291	61.39 (+3.05)
	30%	0.1165	0.2954	0.1789	60.23 (+3.43)
KITTI	100%	0.2248	**0.4510**	**0.2262**	**52.93** (+4.39)
	50%	0.1984	0.4901	0.2917	51.59 (+5.70)
	30%	0.1159	0.5595	0.4436	49.60 (+6.46)

**Table 6:** Expected Calibration Error (ECE, %) and Negative Log-Likelihood (NLL) on the KITTI dataset. The ECE and NLL of the student (SegFormer-B0) are 5.92% and 0.4561, respectively. Bold values indicate the lowest ECE and NLL for each SAFD variant.

	w/o SG		Label		Keywords		Sentence	
Method	ECE	NLL	ECE	NLL	ECE	NLL	ECE	NLL
SAFD-C	3.81	0.4132	3.84	0.4146	**3.44**	**0.4118**	3.47	0.4140
SAFD-S	3.77	0.4150	3.76	0.4186	3.58	0.4146	**3.41**	**0.4129**
SAFD-CS	3.83	0.4140	**3.14**	**0.4110**	3.32	0.4136	3.55	0.4142

## Data Availability

The CamVid and KITTI datasets are publicly available. CamVid is available at https://mi.eng.cam.ac.uk/research/projects/VideoRec/CamVid/ (accessed on 27 July 2026), and KITTI is available at https://www.cvlibs.net/datasets/kitti/ (accessed on 27 July 2026).
